# An image analysis pipeline for automated classification of imaging light conditions and for quantification of wheat canopy cover time series in field phenotyping

**DOI:** 10.1186/s13007-017-0168-4

**Published:** 2017-03-21

**Authors:** Kang Yu, Norbert Kirchgessner, Christoph Grieder, Achim Walter, Andreas Hund

**Affiliations:** 1 Institute of Agricultural Sciences, ETH Zurich, Universitätstrasse 2, 8092 Zurich, Switzerland; 2 Agroscope, Reckenholzstrasse 191, 8046 Zurich, Switzerland

**Keywords:** High throughput field phenotyping, Image analysis, Machine learning, Canopy cover, Image segmentation, Color vegetation index, Light contrast

## Abstract

**Background:**

Robust segmentation of canopy cover (CC) from large amounts of images taken under different illumination/light conditions in the field is essential for high throughput field phenotyping (HTFP). We attempted to address this challenge by evaluating different vegetation indices and segmentation methods for analyzing images taken at varying illuminations throughout the early growth phase of wheat in the field. 40,000 images taken on 350 wheat genotypes in two consecutive years were assessed for this purpose.

**Results:**

We proposed an image analysis pipeline that allowed for image segmentation using automated thresholding and machine learning based classification methods and for global quality control of the resulting CC time series. This pipeline enabled accurate classification of imaging light conditions into two illumination scenarios, i.e. high light-contrast (HLC) and low light-contrast (LLC), in a series of continuously collected images by employing a support vector machine (SVM) model. Accordingly, the scenario-specific pixel-based classification models employing decision tree and SVM algorithms were able to outperform the automated thresholding methods, as well as improved the segmentation accuracy compared to general models that did not discriminate illumination differences.

**Conclusions:**

The three-band vegetation difference index (NDI3) was enhanced for segmentation by incorporating the HSV-V and the CIE Lab-a color components, i.e. the product images NDI3*V and NDI3*a. Field illumination scenarios can be successfully identified by the proposed image analysis pipeline, and the illumination-specific image segmentation can improve the quantification of CC development. The integrated image analysis pipeline proposed in this study provides great potential for automatically delivering robust data in HTFP.

**Electronic supplementary material:**

The online version of this article (doi:10.1186/s13007-017-0168-4) contains supplementary material, which is available to authorized users.

## Background

Modifying and redesigning modern crop varieties to meet the global food and bioenergy demand is a great challenge of contemporary global agriculture [1]. The selection of crops adapted to future climates requires a full understanding of genotype-by-environment interactions (G × E). This urgently requires advanced phenotyping approaches to bridge phenotype-to-genotype gaps, particularly in the field [2]. Although advanced imaging approaches, image processing and computer vision techniques are widely used in plant phenotyping under controlled conditions, they cannot be as easily used in the field [3–5]. Adapting them to be applied in field conditions is a challenging but urgently needed task [6]. Various field phenotyping platforms have been established with the aim of a holistic analysis of crop growth [7–9], but the next challenges consist of image processing, meaningful data extraction, as well as storing and sharing of data [10, 11]. Automation of image processing pipelines will finally facilitate bridging phenomics and genetics towards more powerful genetic analyses and is required to realize the full potential of genome-wide association studies (GWAS) and other modern plant breeding approaches.

In the last decades, most imaging setups for plant phenotyping were established in indoor environments that have well-controlled light conditions (for a review see [12]). Recently, various phenotyping sensors have been equipped on outdoor vehicles and field phenotyping platforms such as mobile phenotyping buggies [9] and stationary platforms [7, 8]. These outdoor platforms, ground vehicles and unmanned aerial vehicles (UAVs) provide new opportunities to promote field phenotyping by routinely deploying sensors and measurements at high spatial and temporal resolution. The goal is therefore to operate sensors under varying natural light conditions, for continuous imaging and quantification of plant growth throughout crop development [12–15]. Also, other important factors such as geometry and location of images as well as movement of camera during image acquisition are among the challenges in field phenotyping.

Appropriate illumination is an important prerequisite for imaging setups under controlled conditions to extract reliable data of phenotypic traits. However, under field conditions, the ever-changing light and weather conditions lead to variable light contrast between upper and lower canopy and between plant and soil. The uncontrollable, weather-related factors lead to enormous difficulties for appropriate image analysis and image segmentation. This in turn constrains the power and throughput of field phenotyping. Computational algorithms have been developed for retrieving quantitative information from images, such as for measuring leaf area, shape and canopy cover [15, 16]. Image segmentation for canopy cover is often based on thresholding methods by setting an appropriate threshold value to distinguish between plants and background [17, 18], or using automatic thresholding methods, such as the Otsu algorithms [16, 19]. Yet often for “poor” images, the use of multiple threshold values is still not sufficient to separate plants properly from soil background. In this context, more sophisticated computational algorithms and machine learning methods have been introduced into plant phenotyping to improve the accuracy of image analysis [20, 21]. One method, based on decision tree classification for canopy cover extraction has already been evaluated for field phenotyping of individual plants at a defined developmental stage [22]. Other studies have already achieved canopy cover segmentation of field images, but have still employed a lot of manual adjustment in segmentation of images [17]. Extracting canopy cover data from a large amount of images taken at various growth stages and weather conditions has not been achieved yet in an automated manner. Such automated approaches are a necessary prerequisite for high throughput field phenotyping (HTFP) approaches that aim at harvesting “big data”.

Therefore, the objectives of this study were to evaluate different methods for retrieving canopy cover data and to tackle the difficulties to achieve high throughput. We attempted to assemble several methods in the framework of a pipeline that allows for a) classifying light conditions, b) quantifying canopy cover dynamics and c) evaluating data quality of the canopy cover and related traits.

## Methods

### Field experiments

A wheat field experiment was conducted at the ETH plant research station Eschikon-Lindau (47.449°N, 8.682°E, 520 m.a.s.l., soil type: varies from calcareous, slightly acidic to gleyic cambisols), Switzerland to study the genotype-by-environment interactions. In the present study, ca. 350 wheat varieties were grown in two growing seasons being harvested in 2014 and 2015 (550 and 700 plots, respectively) to evaluate the conceptual pipeline of image analysis for extracting meaningful canopy cover data for HTFP. The sowing date was 19 Oct 2013 and 20 Oct 2014 for the harvest year 2014 and 2015, respectively, and harvesting date was 5 Aug 2014 and 3 Aug 2015.

### Imaging setup

To capture the canopy development a customized camera holding frame (Additional file 1: Fig. S1, see also [17]) was built to carry a 21 megapixel digital single lens reflector (DSLR) camera (EOS 5D Mark II, Canon Inc., Tokyo, Japan). The camera was commercially customized for monitoring vegetation stress with 3 channels, the visible blue (380–480 nm, B), green (480–560 nm, G) and red (R) that was converted to near-infrared (680–800 nm) (LDP LLD, Carlstadt, NJ, USA, www.maxmax.com). The camera equipped with a Tamron SP 24–70 mm f/2.8 Di VC USD (IF) lens (Tamron Co., Ltd., Saitama, Japan) was mounted onto the frame with a nadir view to the plots from a constant distance of ~2 m to the ground. A fixed focal length at 62 mm was used for imaging. Imaging was performed plot-wise once per day on 33 (7 Nov 2013–16 Apr 2014) and 34 (7 Nov 2014–4 May 2015) measurement dates for the harvest year 2014 and 2015, respectively, with the annually total of 18,216 and 24,276 images.

### Image analysis pipeline and methods

**Fig. 1:**
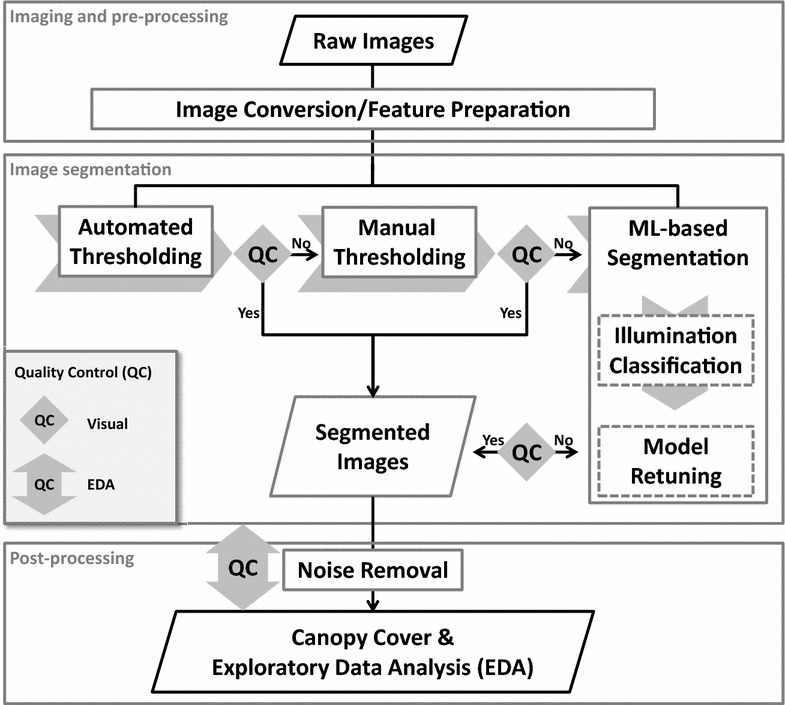
Proposed workflow for an automated image analysis pipeline to classify imaging light conditions, quantify canopy cover dynamics, and assess data quality of traits related to canopy cover

Plant segmentation requires the selection of well suited features and efficient segmentation methods, as well as check of segmentation results that allows to correct and to refine the segmentation process. Therefore, the image analysis pipeline includes three major steps (Fig. 1): (1) image conversion performs the calculations of color spaces and/or color vegetation indices (VIs), (2) image segmentation implements classifications based on thresholding and/or machine learning (ML) as described in the following sections, and (3) post-processing includes the removal of noise in the segmented images, quality control (QC) by visual inspection and the exploratory data analysis (EDA) for identifying potential bias and outliers.

**Fig. 2:**
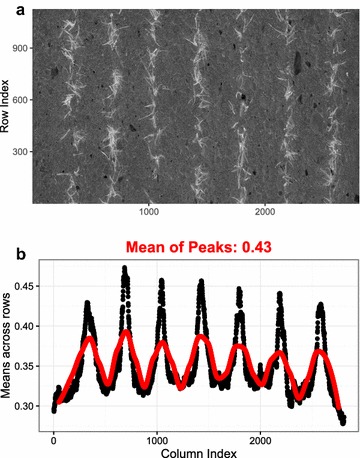
Automated thresholding on a calculated NDI3 image (**a**) by averaging across image rows (**b**, n = the size of image height). *Black dots* show the row-mean values along with the image column index, and *red line* is the smoothed spline indicating the positions for peaks and troughs that are detected for the row-means (μRow)

Thresholding methods can be grouped into automated and manual procedures. Manual thresholding methods often use grayscale images and VI images derived from original images. Examples for color VI images are the Excess Red (ExR), Excess Green (ExG) and Normalized Difference Index (NDI) images [16, 17]. In this study, automated thresholding was performed using Otsu’s method [23] and/or an image row-means (μRow) based method. We also tested multiband thresholding. However, we have not been successful in automating the procedure for the large amount of images to be processed for our study, and here multiband thresholding is not considered. The μRow method was proposed based on the specific patterns in the images of row crops (Fig. 2a), where plant rows could be detected by determining the peaks in the mean values that were averaged across all the rows of an image matrix (Fig. 2b). The threshold then could be defined as the mean value of the detected peaks.

In order to evaluate the capability of the use of different color components and VIs in thresholding, images were converted to ExR (ExR = 1.4R-G) and its blue channel variant ExB (ExB = 1.4B-G) [16], two-band NDI (NDI2 = (R − B)/(R + B)) and three-band NDI (NDI3 = (R + G − 2B)/(R + G + 2B)) images [24], as well as the products of color components and VIs such as NDI2*a (a: a-channel in the Lab color space), NDI3*a and NDI3*V (V: V-channel in the HSV color space). Subsequently, the Otsu and μRow thresholding were implemented to segment the different VI images, and the VI providing the best separability would be used as an additional predictor in the ML-based classification models.

ML methods fall into two broad categories: unsupervised learning and supervised learning, both of which were applied in this study for image segmentation. An unsupervised machine learning approach based on the K-means clustering was implemented by first determining 3 clusters of *a* and *b* color channels of the Lab color space (CIE 1976 L*a*b*, see [25]) and then selecting the cluster with the highest NDI3 values as the cluster related to plants (similar to the construction of NDVI, see details in [24]). A supervised machine learning approach based on the decision tree (DT) segmentation model (DTSM) [22] and support vector machines (SVM) was implemented. Nine color components including the R (red), G (green) and B (blue) in the RGB color space; H (hue), S (saturation) and V (value) in the HSV color space; and L (lightness), *a* and *b* (color-opponent dimensions) in the Lab color space [25] and the NDI3*V (product of NDI3 and V) were used to classify each pixel into two classes, background and foreground (plants). 150 images were selected for training, in which a total number of 2,909,000 pixels were marked as the training data. The training data was collected using the software EasyPCC [22], which allows to interactively mark lines on plants and background and then saves pixel-based records and output as a txt-file.

To cope with highly heterogeneous illumination variations, an imaging illumination classification method based on the support vector machines (SVM) was proposed to classify high light-contrast (HLC) and low light-contrast (LLC) images. Here, based on illumination differences, we define an image as HLC image when extremely bright and dark regions/pixels observed in the image, whereas defined as LLC image when all details of scene are clearly captured in the image. Extreme bright and dark regions are identified by visual inspections on the images and image histograms. Importantly, the HLC and LLC images definition is different from the high/low contrast photography technique. Three image exposure intensity features consisting of the histograms of R, G, and B channels were used to classify images into two classes, HLC and LLC images. The numerical distribution of the histogram of each channel was calculated in 256 bins, and thus a concatenated vector of 256*3 numbers was constructed for each image in the SVM-based illumination classification model. Accordingly, in the following step, ML-based segmentation models employing the DT and SVM algorithms were trained for the two illumination classes individually to compare their performance under different illuminations, i.e., models for HLC (M_HLC_) and LLC (M_LLC_) images, respectively, as well as general models for all light conditions (M_ALC_).

Prior to the final calculation of canopy cover, “salt & pepper noise” removal was performed to all of the segmented images by applying a median filter (5 × 5 size) [26] and removing objects smaller than 400 pixel size (also see functions indicated in Fig. 1). To control the quality of the segmentation of thousands of images visually requires tremendous efforts. In time series measurements performing the exploratory data analysis (EDA) on the extracted canopy cover data can help to identify critical time points with bias of segmentation. The plot-based canopy cover vector of one date is correlated with the vector of any other date. Low correlation coefficients indicate low consistency, which might be caused by segmentation errors or rapid changes in the ranking of genotypes. The ranking change is attributed often to physiological or environmental changes—for instance snowing and snow melting during the winter. In this study, EDA including the correlation analysis were implemented in the R software [27]. Image processing, ML-based models and the one-way ANOVA test of model performance were implemented in Matlab (The MathWorks, Inc., Natick, MA, USA).

### Segmentation accuracy

Segmentation accuracy was evaluated with three quality factors: *Qseg* [16], *Sr* [22] and an error factor *Es*, which are given as Eqs. (1), (2) and (3), respectively,

1 $$Q_{seg} = \frac{{\sum\nolimits_{k,j = 0}^{k,j = n,m} {\left( {S(i)_{k,j} \cap R(i)_{k,j} } \right)} }}{{\sum\nolimits_{k,j = 0}^{k,j = n,m} {\left( {S(i)_{k,j} \cup R(i)_{k,j} } \right)} }}$$

2 $$Sr = \frac{{\sum\nolimits_{k,j = 0}^{k,j = n,m} {\left( {S(i)_{k,j} \cap R(i)_{k,j} } \right)} }}{{\sum\nolimits_{k,j = 0}^{k,j = n,m} {R(i)_{k,j} } }}$$

3 $$Es = \frac{{\sum\nolimits_{k,j = 0}^{k,j = n,m} {S(i)_{k,j} \cap R(!i)_{k,j} } }}{{\sum\nolimits_{k,j = 0}^{k,j = n,m} {R(i)_{k,j} } }}$$

where *S* is the set of segmented plant (i = 1) or background pixels S(i = 0) separated by a thresholding or ML-based method, *R* is the reference set of ‘manually’ segmented plant *S*(i = 1) or background *S*(i = 0) pixels separated by hand-click in Adobe^®^ Photoshop^®^ CS6 (Adobe Systems Inc., San Jose, CA, USA). Indices *k, j* are the row and column coordinates of an image, respectively, and *n, m* are the width and height of the image size, respectively. Separation accuracy is based on logical operators: ∩ (logical and), ⋃ (logical or) and ! (logical not), compared on a pixel by pixel basis of target image *S* to the reference image *R*. *Qseg* ranges from 0 to 1 and measures the consistency between *S* and *R* on a pixel-by-pixel basis; the value 1.0 represents a perfect segmentation [16]. Similarly, *Sr* measures the consistency within the image region of plant pixels [i.e., R(i = 1)] [22], whereas *Es* indicates the portion of misclassified plant pixels (true background) relative to true total plant pixels [i.e., R(i = 1)]. The decomposed computation for the three quality measures using segmentation masks is described in the Additional file 1. Validation of segmentation accuracy of different methods was performed by analyzing a validation set of manually-delineated images containing 20 HLC and 20 LLC images and comparing the *Qseg*, *Sr* and *Es*.

## Results and discussion

### Comparing different VI images for threshold segmentation

**Fig. 3:**
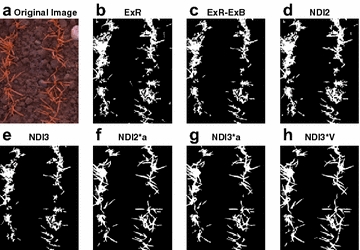
Images shown are the original image (**a**) and segmented images based on seven different vegetation indices (**b**–**h**), ExR, ExR –ExB, NDI2, NDI3, NDI2*a, NDI3*a, NDI3*V. Segmentation was performed via automated thresholding based on the image row-means (μRow) method. For fine display only a region-of-interest (ROI) is presented

**Fig. 4:**
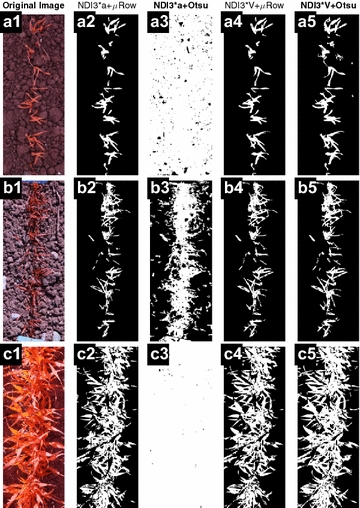
Comparison of using two automated thresholding methods (μRow and Otsu) on the NDI3*a and NDI3*V images for canopy cover segmentation. Images in column 1 are the original images (**a1**, **b1**, **c1**) that were taken under different illumination conditions with the modified DSLR camera. For fine display only small region-of-interests (ROIs) are presented

Choosing proper VIs is the key step for thresholding. Images of several VIs were converted from the original images, and their threshold values were determined using the μRow method. The VI images were then segmented according to the μRow-based thresholds (Fig. 3). Results showed that the ExR, ExR-ExB, NDI2 and NDI3 images produced comparable results, where the threshold values appeared to be either too rigorous or unable to separate the plant from background pixels (Fig. 3b–e). Generally, NDI2*a, NDI3*a and NDI3*V allowed for efficient segmentation compared to the aforementioned four VIs. The a- and V-component enhances the differences between plant and background, where the differences are small in the VI images. For the high illuminated HLC images, however, the potential is limited (Fig. 4). Furthermore, the NDI3*V produced the best segmentation among all of the VI images, which was confirmed based on visual inspection on a subset of images.

### Comparing μRow and Otsu for threshold segmentation using NDI3*a and NDI3*V

According to the comparison for the general performance of different VIs, the best performing two indices (NDI3*a and NDI3*V) were further evaluated for automated thresholding. NDI3*a and NDI3*V images were calculated for three original images (Fig. 4a1, b1 and c1), and μRow and Otsu methods were used to determine the threshold and segment the images. Results showed that the μRow method allowed for the determination of proper threshold values and segmentation on NDI3*a (Fig. 4a2, b2, c2) and NDI3*V images (Fig. 4a4, b4, c4). In contrast, Otsu did not perform constantly on NDI3*a and NDI3*V images, and it allowed proper segmentation only on the NDI3*V images (Fig. 4a5, b5, c5). By incorporating brightness differences into the NDI3 images, the V component of the HSV color space enabled to properly determine threshold for NDI3*V, which applies particularly to field-based phenotyping [18, 28]. However, field illuminations cannot be easily controlled and strong light contrast often causes saturated pixels and regions where VI-based image transformations could not significantly enhance the differences between plants and background. In this case, simple thresholding methods are more likely to induce a systematic error of decrease or increase (Fig. 4b, c) in the segmented area, and QC appears to be particularly difficult and laborious if one relies on visual inspection and judging of errors.

### Influence of different imaging time (illumination) on segmentation

The μRow and Otsu methods were able to determine image-specific threshold values given that optimal VI was employed, allowing for automated threshold determination for canopy cover segmentation. However, the capacity of these methods is limited by the choice of VIs and/or color components for thresholding. In contrast, ML-based methods that use more features of different color components and VIs might be more applicable for varying imaging conditions in the field. Thus, we evaluated whether the ML-based segmentation is independent of imaging time in HTPP in comparison with manual segmentation.

**Fig. 5:**
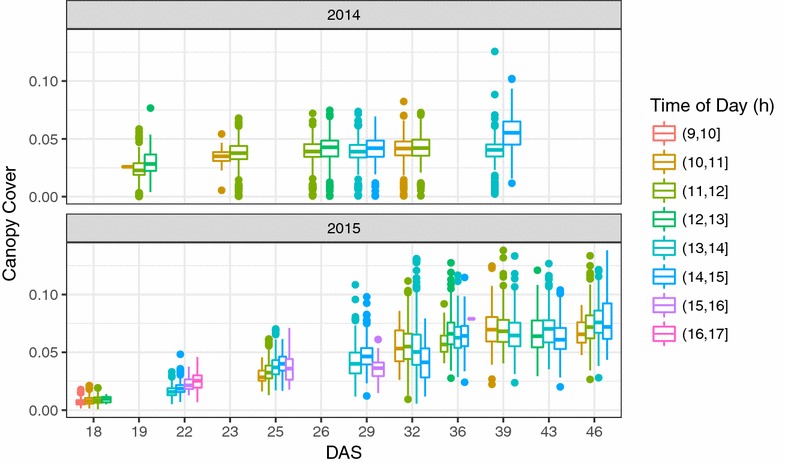
Canopy cover plotted as a function of imaging time/illumination of the day. Canopy cover shown here was extracted from ca. 10,000 images during the growth period DAS (days after sowing) 18–50 in 2014 and 2015. Manual thresholding and DTSM methods were used for segmentation in 2014 and 2015, respectively

The effect of different illuminations on the results of image segmentation was evaluated by analyzing ~16,000 and ~25,000 images in 2014 and 2015, respectively, using manual thresholding (see details in [17]) and DTSM models [22]. The images were taken on different days during early canopy cover development in 2014 and 2015. Normally, 2–3 h were needed for imaging all of the ~700 plots on each measuring day. During the course of the imaging, light conditions sometimes changed significantly and thus induced systematic variability and errors in the extracted canopy cover. For example, there was an increasing trend at DAS (days after sowing) 39 in 2014 as illustrated in Fig. 5. There was no strong trend along with the imaging time on other measurement days, compared to the increase in canopy cover variations with increasing DAS. Results showed that both the thresholding and ML methods were affected by the differences in imaging time/illumination.

### Pre-classification of imaging conditions

**Fig. 6:**
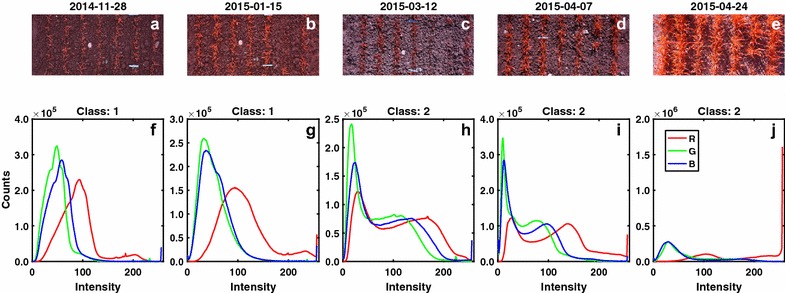
Raw images (**a**–**e**) and their classification results of image illumination conditions (**f**–**j**) based on the image histograms of the R, G and B channels. The Classes 1 and 2 stands for the low light-contrast (LLC) and high light-contrast (HLC) conditions, respectively

To account for strongly differing illumination, it was decided to classify images into HLC (class 1) and LLC (class 2) situations by applying the SVM illumination classification model. LLC images yielded the majority of pixels located in the low to medium intensity range in all of the three channels (Fig. 6a, b), whereas less pixels were observed in the range of high intensities in the three channels. In contrast, HLC images had a large portion of pixels distributed in the range of high intensities compared to LLC image, and therefore the former often had over-exposed pixels or regions in the image (Fig. 6e). The trained illumination classification model was validated again on the independent set of 40 reference images (20 HLC and 20 LLC images). The model could correctly classify the illumination classes for 35 out of the 40 images (see details of classification results in the Additional file 1: Figs. S2 and S3).

**Fig. 7:**
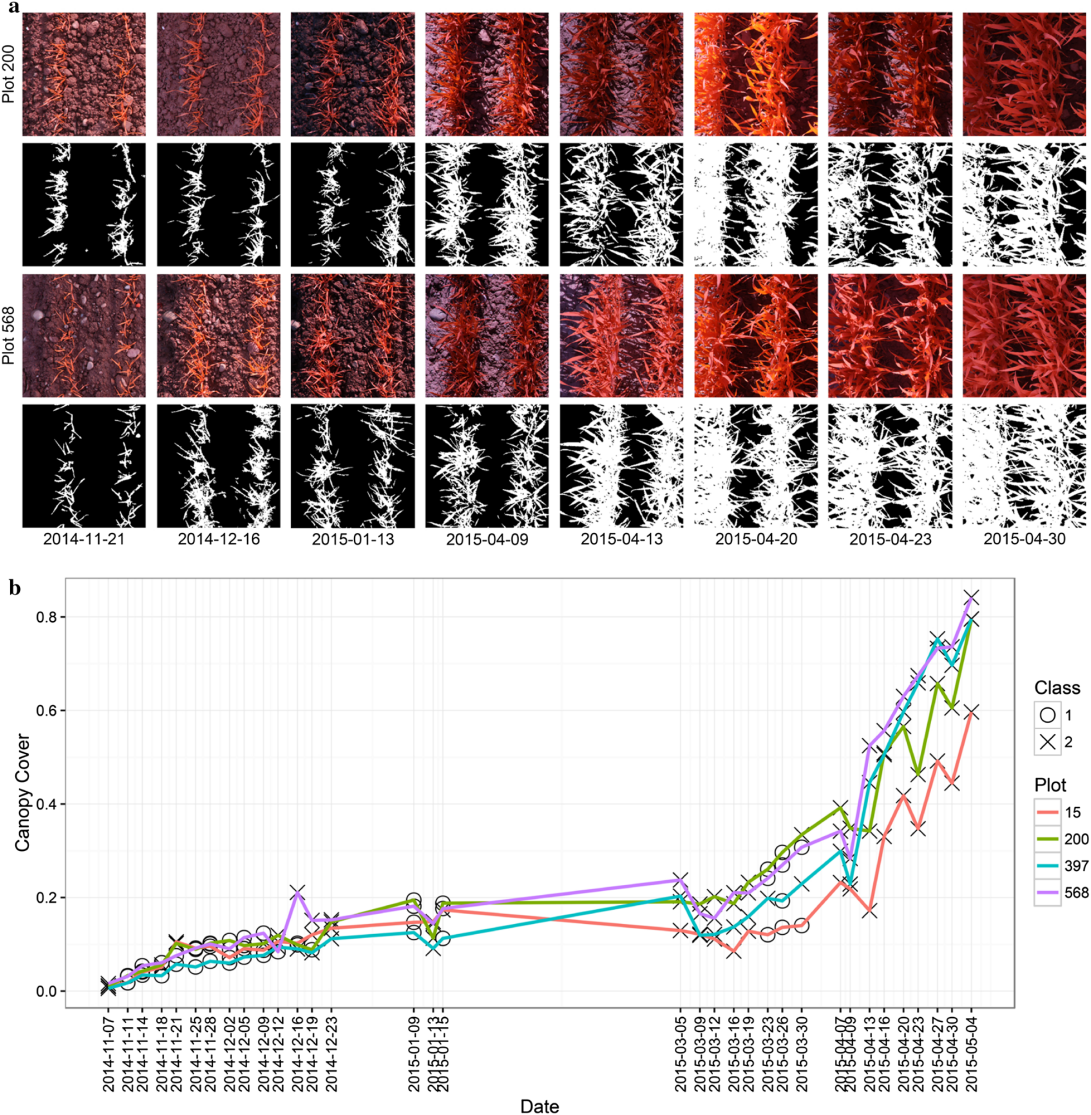
The segmentation of images for one wheat genotype (CLARO) that was randomly placed on the experimental field, with plot number No. 15, 200, 397 and 568. Original and their segmented images on 8 dates are displayed (**a**), and their predicted illumination classes labeled (**b**). The segmentation of class 2 images resulted in canopy cover values that caused discontinuities in the chronological depiction of canopy cover development more often than in the case of class 1 images (**b**). Yet, during such discontinuities the ranking of genotypes often was the same as in preceding and subsequent measurement dates (B, see values throughout April). The time gaps following 2014-12-23 and 2015-01-15 indicate the periods of snow cover. For fine display only small region-of-interests (ROIs) are presented

Segmentation was performed on all measurement dates in 2015 using the same DTSM model, and the results showed that the illumination had a significant effect on the segmentation and resulting canopy cover as revealed by the time series patterns (Fig. 7). The segmentation of class 2 images resulted in canopy cover values that caused discontinuities in the chronological depiction of canopy cover development more often than in the case of class 1 images (Fig. 7b), which was also observed in the EDA step of the pipeline by applying the correlation analysis between different dates (next sections). Yet, also during such ‘discontinuities’ the ranking of the four plots representing the same genotype often was the same as in the preceding and subsequent measurement dates (see e.g. analyses throughout April in Fig. 7b). From the perspective of plant physiology, the canopy cover should not decrease dramatically during stem elongation, and thus data smoothing methods can be used to compensate for the segmentation error due to the high illumination. The observed discontinuities could be also attributed to reactions of plants to sudden environmental changes, for example snow melting often led to reduced canopy cover in the following period (see the time gaps in Fig. 7).

### Comparison of image segmentation models of different illuminations

**Fig. 8:**
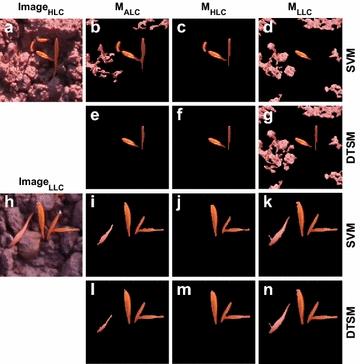
Comparison of the original (**a**, **h**) and segmented images (**b**–**g**, **i**–**n**) on two different illumination days using the three illumination-scenario specific models: general model for all light conditions (M_ALC_) and models for high light-contrast (M_HLC_) and low light-contrast (M_LLC_). For each scenario, the support vector machines (SVM) and decision tree segmentation model (DTSM, see Guo et al. [22]) were used for training. See the details of the segmentation accuracy in the Additional file 2, the image 2014_11_07_FWW0070706

Based on the classification results of image illuminations, the three types of ML-models (M_ALC_, M_HLC_ and M_LLC_) were applied for the classification of a subset of images for quantitative comparison. Based on visual comparison (Fig. 8), for LLC images the M_LLC_ model enabled a more precise segmentation than the M_HLC_ model (Fig. 8j, k, m, n). Likewise, the M_HLC_ model yielded more precise segmentation than the M_LLC_ model when applied to the HLC images (Fig. 8c, d, f, g). The general model (M_ALC_) did not significantly improve the segmentation accuracy, although the two identical training sets for the M_HLC_ and M_LLC_ models were pooled for training the M_ALC_ model (see Additional file 2: Table S1). As expected, the M_ALC_ model yielded better segmentation for HLC images (e.g. Fig. 8a) than did the M_LLC_ model (Fig. 8b, d, e, g). Likewise, for the LLC images (e.g. Fig. 8h) the M_HLC_ model did not outperform the M_ALC_ model (Fig. 8i, j, l, m). Results suggest that the M_HLC_ and M_LLC_ were tailored to the specific training data (illumination differences) compared to the M_ALC_ model.

**Fig. 9:**
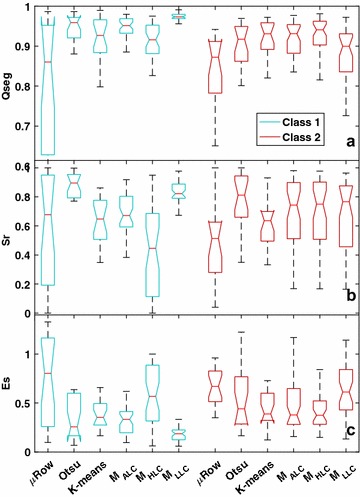
Boxplots of three quality measures, Qseg (**a**), Sr (**b**) and Es (**c**), showing the performance of for canopy cover segmentation using the μRow, Otsu and three types of ML-based models (M_ALC_, M_HLC_, M_LLC_ models using DT and SVM algorithms), which were evaluated on 20 LLC images (Class 1) and 20 HLC images (Class 2). Non-overlapped box notches indicate significant differences between the segmentation methods, with 95% confidence. The 40 reference images for validation are publicly available in the ‘figshare’ repository [30]

We validated the performance of all the ML-models by calculating the quality measures (Eqs. 1–3) and comparing with the μRow and Otsu thresholding based on the 40 segmentation reference image (manually segmented images, see Additional file 2 for a list of results). For the segmentation of LLC images, M_LLC_ models yielded very high accuracy (*Qseg* and *Sr*) and low error (*Es*) compared to μRow, Otsu, K-means and M_ALC_ and M_HLC_ models. ANOVA test showed that significant differences in *Qseg*, *Sr* and *Es* were observed between the M_ALC_, M_HLC_ and M_LLC_ models for the LLC images, highlighting that M_LLC_ models outperformed the former two models. In contrast, M_HLC_ models generally yielded high accuracy (*Qseg* and *Sr*) and low error (*Es*) for the HLC images (Fig. 9) that were taken under HLC conditions (e.g., images in Fig. 6c–e). For HLC images, the improvements of M_HLC_ models relative to the M_ALC_ and M_LLC_ models were not statistically significant (Fig. 9), which might be attributed to the limited data set of test images or to that oversaturated pixels provided inefficient information for learning. Although the M_ALC_ model employed the pooled training data from both HLC and LLC images, it showed relative low segmentation accuracy compared to the M_LLC_ and M_HLC_ models. Results suggest the benefit of pre-classification of illumination differences and the potential for improving accuracy with the illumination-specifically trained models. Notably, the μRow method yielded large variability in the *Qseg*, *Sr* and *Es*, which was caused often by poor plant-row detection, e.g., no clear pattern of plant rows is observable when images were taken shortly after germination.

Similar to the results in a previous study in which Guo et al. [22] applied DTSM to improve the segmentation for images of individual plants, HLC images increased the difficulty for segmentation compared to LLC images. Furthermore, our study demonstrated at plot level that integrated ML-based models were able to significantly improve the segmentation accuracy compared to simple thresholding methods. This improvement depends on a high resolution of the images; when the resolution is too low, the probability increases that individual pixels contain mixtures of plant and soil, making the approach less powerful.

The appropriate segmentation of wheat plants with their narrow leaves is more difficult compared to other species that have wider and bigger leaves. Narrow leaves are prone to be divided in the segmentation because oversaturated pixels occurred in the middle of a leaf (Fig. 7a). Also dirty leaves due to dislodged soil particles after heavy rains are difficult to segment (Fig. 6). In addition, randomly stacked leaf layers limit the potential of using shape-based features and methods in HTFP [29].

Ideally, a general model being able to precisely segment all the images will simplify the image analysis pipeline in HTFP [20, 22]. Although the optimal approach might be the use of a general model for all different light conditions, its training often demands more computational power than the training of several scenario-specific models (see Additional file 1: Fig. S4). Training models according to distinct illumination conditions could significantly reduce the costs in time as well as the computational power needed for heavy model training (Additional file 1: Fig. S4), and thus accelerates the progress and throughput for data post-processing of HTFP.

### Exploratory data analysis (EDA) for identifying potential bias and outliers

**Fig. 10:**
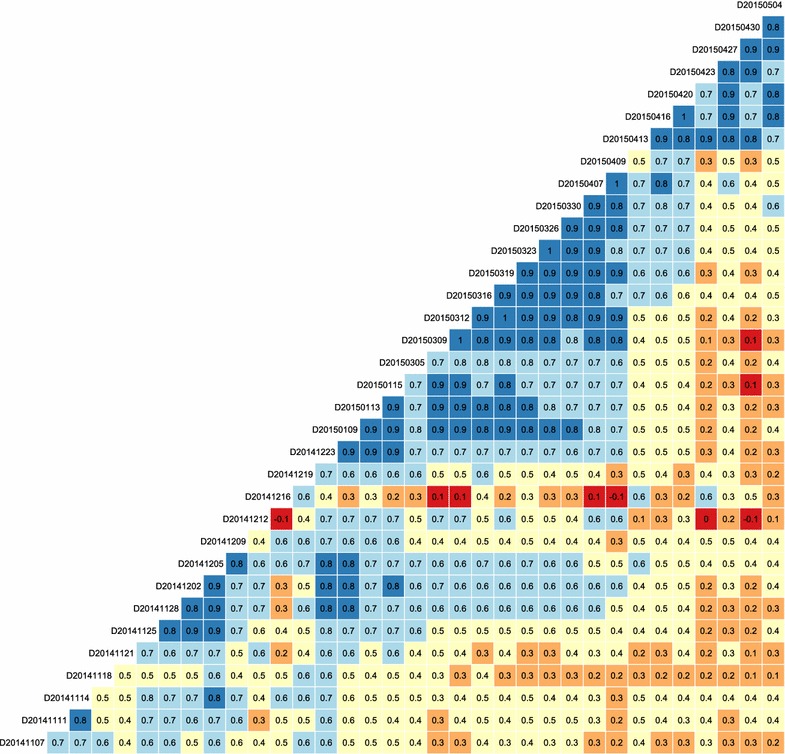
Correlation (r) matrix showing the correlations of canopy cover between different measurement times for an entire growing season. *Labels* on the diagonal indicate the dates of measurements

Quality control is a critical step following segmentation in the whole image analysis pipeline (Fig. 1). To evaluate the quality of the segmentation (Fig. 1) on individual days, we performed correlation analysis for the canopy cover (extracted using a DTSM general model) of the successive measurement days. Results showed that canopy cover between successive measurement days was highly correlated, except for the measurement day December 12, 2014 (D20141212, Fig. 10). The correlation between this day with its previous (D20141209, r = 0.4) and following (D20141216, r = 0.1) days were lower than the ones between other successive measurement days (Fig. 10), which confirms the observed large deviations in one of the experiment plots (see Plot 568 in Fig. 7b). As expected, the images for the December 12, 2014 were of very high light contrast compared to images for the previous and following days (Additional file 1: Fig. S5). To lower the bias of phenotyping results and interpretation of G × E interactions, the data acquired under such conditions could be excluded in further analysis. Importantly, EDA such as the correlation analysis presented here allowed for evaluation of potential segmentation errors and could help define necessary training data and fine tune segmentation models.

### Challenges and opportunities for an automated image analysis pipeline for HTFP

The three steps proposed in the image analysis pipeline (Fig. 1) are in line with the main challenges for analyzing images in HTFP. For instance, canopy cover segmentation depends on proper VIs/color components and other potential features to achieve reliable segmentation, which is critical for both thresholding and machine learning methods. However, among other influence factors, illumination variability within an image and/or between images affects the segmentation strongly, particularly when imaging a large amount of breeding lines in the field. Interestingly, results showed that a pre-classification of imaging illumination condition could improve the final segmentation. In addition to the aforementioned two challenges, in a normal breeding program the volume of data generated by HTFP over multiple days and years makes “eyeball”-based QC difficult. Thus, automated control of segmentation quality is vital. Our results highlight the potential VIs/features and alternative ML-models for accounting for field illumination variability as well as a simple strategy of EDA in the developing of an automated image analysis pipeline for HTFP.

Nevertheless, the use of a general model is essential and it is often necessary even if the model performance is not optimal, particularly for HTFP that needs to extract data from a large amount of images in a timely manner. A general model can be used to generate the first results, which is critical for appropriately determining scenario-specific models such as a model trained for images of flooded fields or wet plants. Also, a quality indicator can be incorporated in the first results – for instance, image specific flags for illumination conditions could serve as a filter when evaluating the results (Additional file 1: Fig. S6). This study focuses only on two scenarios of illumination differences, which can be further improved and integrated into imaging platforms for automatically identifying illumination conditions and for selecting appropriate analysis methods. Concerning the case of low segmentation quality of canopy cover and obtaining data is crucial for certain growth phases, specific models might need to be trained using data on certain dates in the study (e.g. low r values in Fig. 10). By considering the two scenarios and applying the proposed image analysis pipeline, we were able to improve the correlation between the canopy cover and growing degree days (Additional file 1: Fig. S7), demonstrating the capability of imaging based high throughput phenotyping for characterizing plant growth dynamic in response to the seasonal temperature cumulation.

High dynamic range (HDR) photography could be applied for field phenotyping as there are commercial products that support HDR imaging on the market. “Digital darkroom” also makes HDR imaging feasible provided that threefold or more storage space and time for exposure are available. Automation of QC remains to be a challenge following the image processing in HTFP, and integrated methods and platforms for imaging, image processing, machine learning and data science are needed to extract reliable data in HTFP [6, 8, 9].

## Conclusions

Timely extracting meaningful data from a very large amount of high resolution images is the bottleneck in high throughput field phenotyping (HTFP); therefore the development of advanced image analysis pipelines is imperative. This study has established an image analysis pipeline for HTFP of wheat canopy cover development. It attempts to tackle the difficulties encountered from image analysis till the delivering of reliable phenotypic data on a plot basis. A data set of more than 40,000 images collected throughout two growing seasons was used to evaluate the pipeline. We found that the NDI3*V and NDI3*a indices in combination with automatic thesholding using μRow and Otsu methods allowed for appropriate separation of wheat plants and background compared to other VIs evaluated in this study. Significant improvement was further achieved by applying illumination-specific models based on machine learning, which improved the accuracy and lowered the computing time. EDA analysis was able to assist the quality control of image segmentation by examining temporal correlation changes in the time series of extracted canopy cover. The proposed image analysis pipeline enabled to extract the canopy cover time series from canopy images at high throughput, and it can be adjusted for imaging-based phenotyping of other traits and species in HTFP.

## Additional files

**Additional file 1.** Supplementary information for the segmentation quality measures, imaging setup for canopy cover measurements, image illumination classification and its effect on segmentation results, differences in model training time as well as segmentation improvement through the proposed image analysis pipeline.

**Additional file 2.** List of segmentation accuracy measures for six different methods when applied to 40 reference images.
