# Supplementary material for: An image analysis pipeline for automated classification of imaging light conditions and for quantification of wheat canopy cover time series in field phenotyping
Source: Plant Methods. 2017 Mar 21;13:15. doi: 10.1186/s13007-017-0168-4 (PMC5361853; doi:10.1186/s13007-017-0168-4)
Supplement: Supplementary file 1 — Additional file 1. Supplementary information for the segmentation quality measures, imaging setup for canopy cover measurements, image illumination classification and its effect on segmentation results, differences in model training time as well as segmentation improvement through the proposed image analysis pipeline. [file 13007_2017_168_MOESM1_ESM.pdf]

Additional file 1

Three segmentation quality measures described in the way of calculation of pixel counts based on segmentation and segmentation reference masks:

$$Qseg = \frac{S_{plant} \cap R_{plant} + S_{soil} \cap R_{soil}}{R_{plant} + R_{soil}}$$

$$Sr = \frac{S_{plant} \cap R_{plant}}{R_{plant}}$$

$$Es = \frac{S_{plant} \cap R_{soil} + S_{soil} \cap R_{plant}}{R_{plant}}$$

Here, the S and R denote the computer segmented image and reference segmentation image.

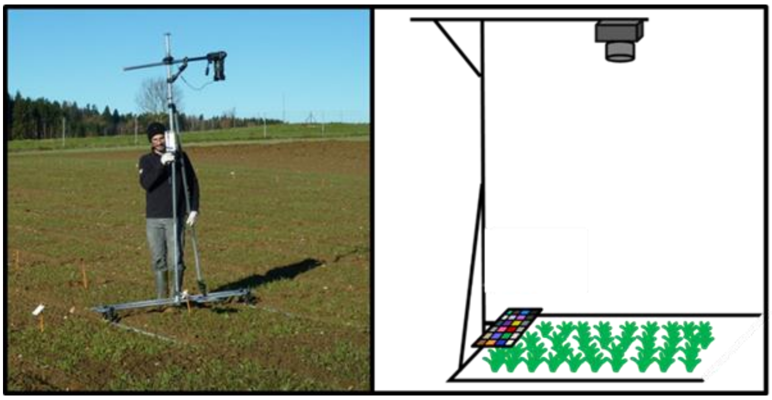

Fig. S1. Imaging setup for measuring canopy cover using a DSLR camera mounted on a custom-made aluminium frame. The linear distance between the camera and ground is ~2 m.

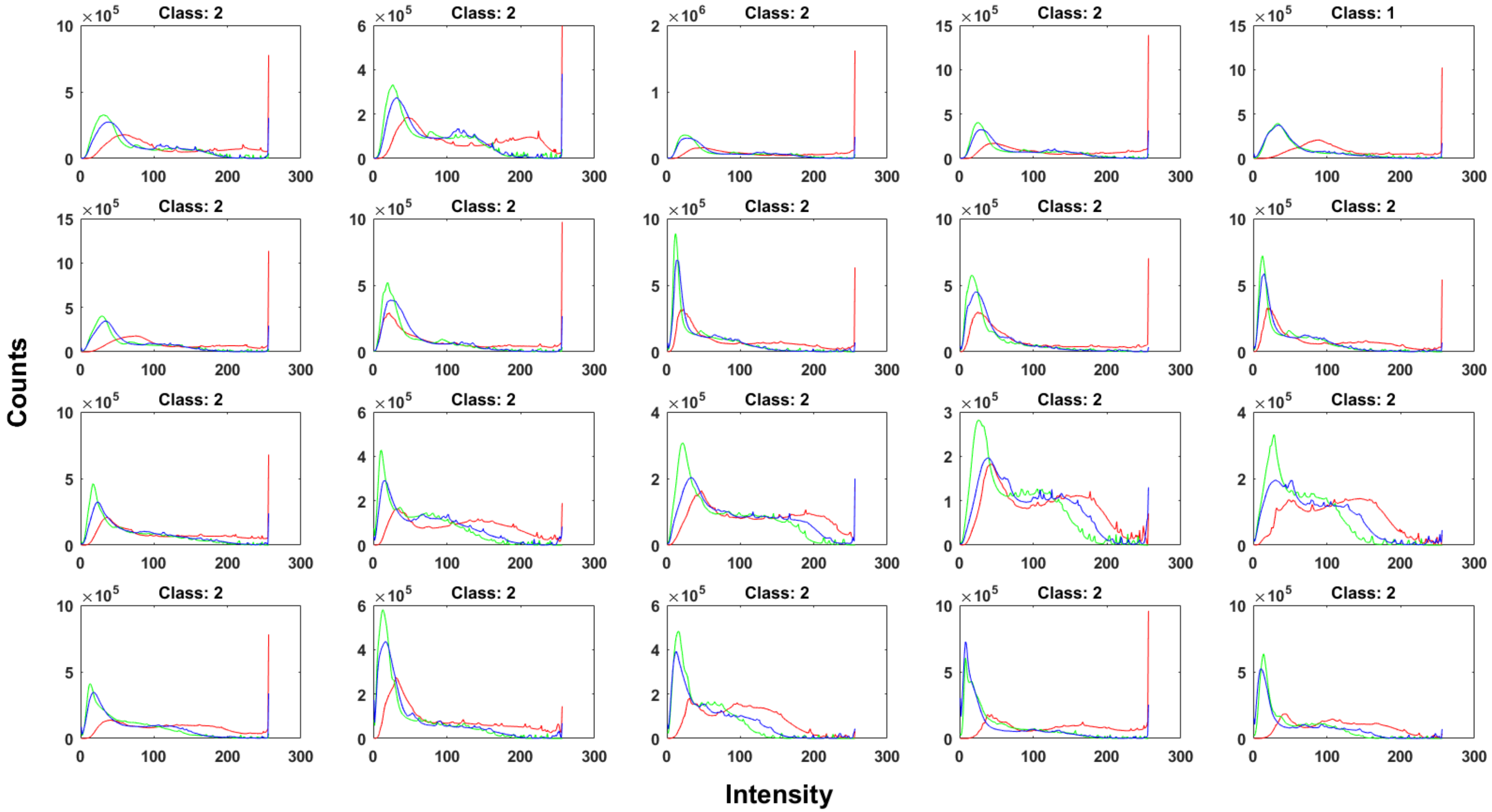

Fig. S2. SVM-based illumination classification on the new data set of 20 HLC images (Class 2). The model correctly predicted the light conditions for 19 images, with an accuracy of 95%.

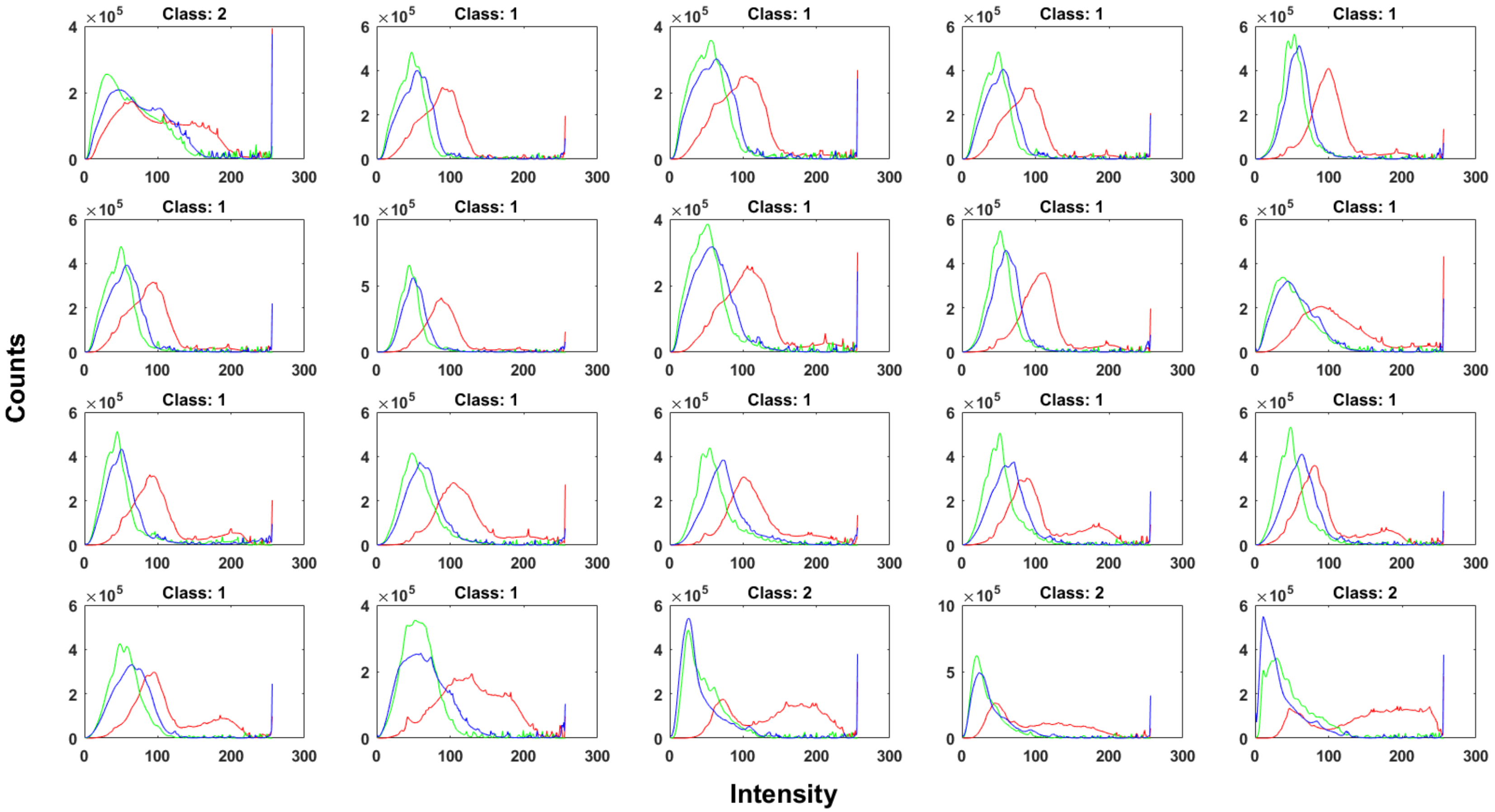

Fig. S3. SVM-based illumination classification on the new data set of 20 LLC images (Class 1). The model correctly predicted the light conditions for 16 images, with an accuracy of 80%.

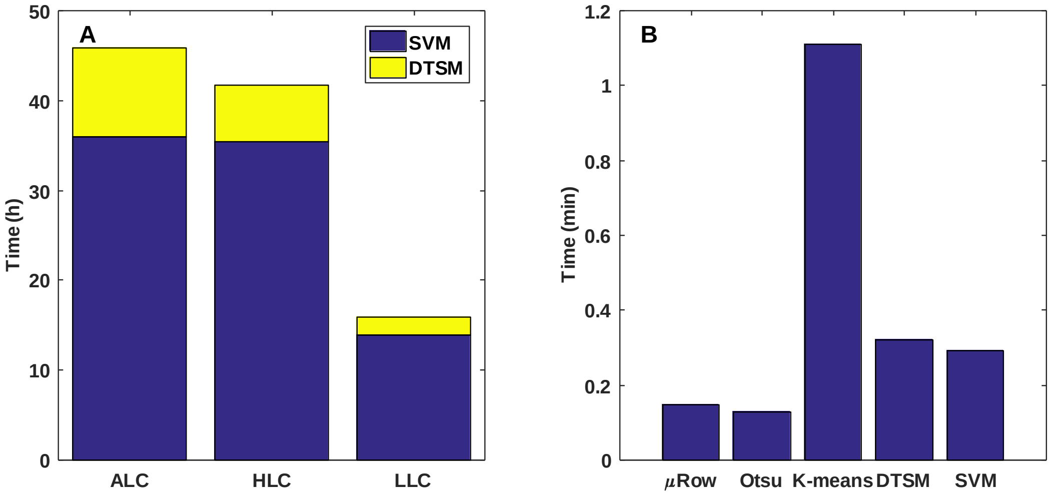

Fig. S4. Training time (hours) for the three types of models for ALC, HLC and LLC images (A) based on DT and SVM methods, and they were compared with µRow, Otsu and K-means clustering in computing time (minutes) for segmentation of one image (B).

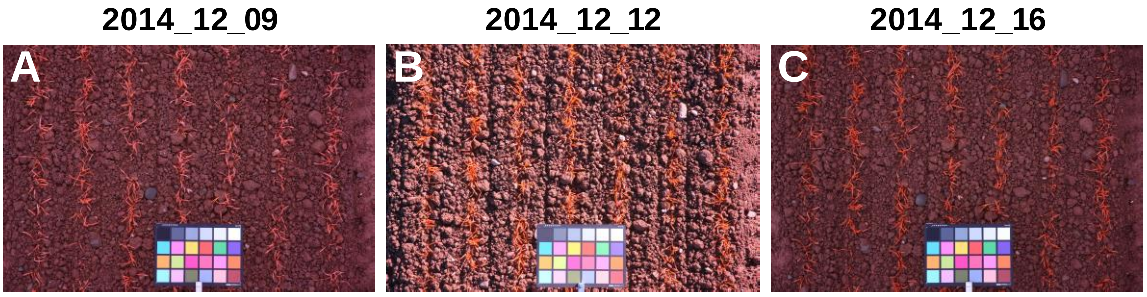

Fig. S5. Images taken on three different days under different light condtions. The canopy cover extracted from images for the day 2014-12-12 (B) yielded low correlations with the (A) previous and (C) following days.

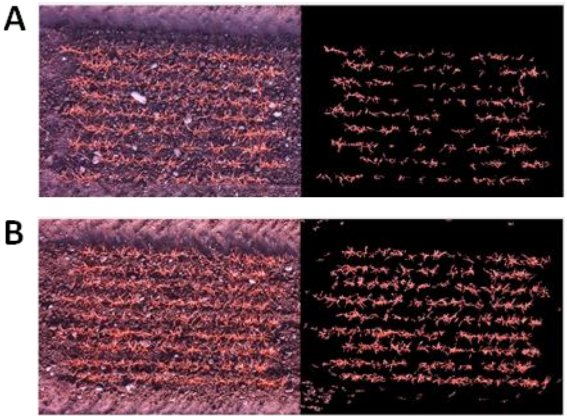

C

Image	CC	Illumination
A	0.06	1
B	0.14	2

Fig. S6. Image specific flags for illumination conditions could be used as a metadata item and serve as a filter when evaluating the canopy cover results in high throughput field phenotyping.

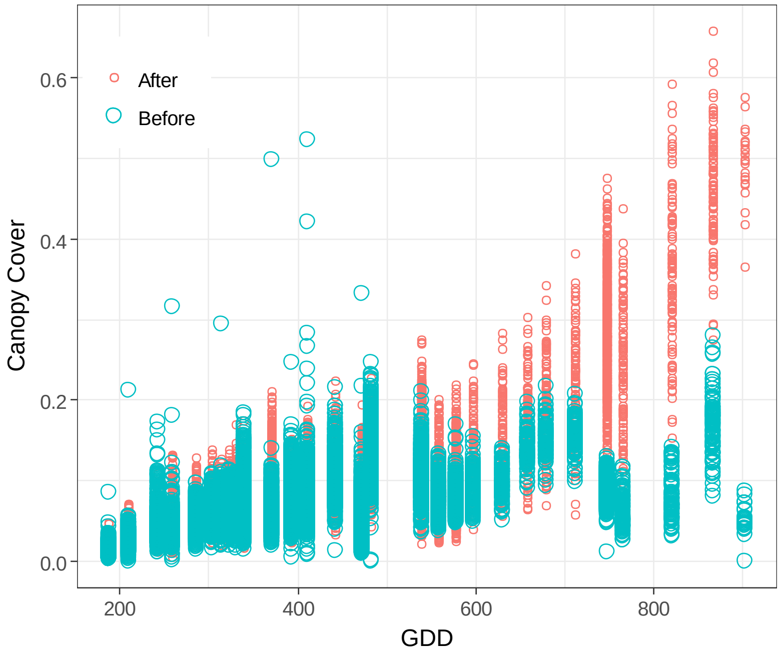

Fig. S7. Comparison of the segmentation results for canopy cover in relation to growing degree days (GDD) before and after the use of the proposed image analysis pipeline.
