# Supplementary material for: An image analysis pipeline for automated classification of imaging light conditions and for quantification of wheat canopy cover time series in field phenotyping
Source: Plant Methods. 2017 Mar 21;13:15. doi: 10.1186/s13007-017-0168-4 (PMC5361853; doi:10.1186/s13007-017-0168-4)
Supplement: Supplementary file 2 — Additional file 2. List of segmentation accuracy measures for six different methods when applied to 40 reference images. [file 13007_2017_168_MOESM2_ESM.pdf]

Additional file 2

Table S1. Segmentation accuracy measured by Qseg, Sr and Es for 6 different methods. Results were validated based on 40 images. For each image the performances of segmentation methods is ranked based on the Qseg values in a descending order.

Class	Image	Method	Model	Qseg	Sr	Es
1	2014_11_07_FWW0070706	DTSM	LLC	0.9817	0.8145	0.2393
1	2014_11_07_FWW0070706	SVM	LLC	0.9801	0.7805	0.2602
1	2014_11_07_FWW0070706	K-means	K-means	0.9782	0.7907	0.2856
1	2014_11_07_FWW0070706	Otsu	Otsu	0.9781	0.8096	0.2868
1	2014_11_07_FWW0070706	DTSM	ALC	0.9706	0.6330	0.3850
1	2014_11_07_FWW0070706	SVM	ALC	0.9694	0.6044	0.4008
1	2014_11_07_FWW0070706	DTSM	HLC	0.9643	0.5562	0.4675
1	2014_11_07_FWW0070706	SVM	HLC	0.9568	0.4515	0.5651
1	2014_11_07_FWW0070706	Rowmeans	Rowmeans	0.5066	0.9994	6.4563
1	2014_11_11_FWW0070001	SVM	LLC	0.9909	0.8022	0.1996
1	2014_11_11_FWW0070001	DTSM	LLC	0.9900	0.7865	0.2183
1	2014_11_11_FWW0070001	K-means	K-means	0.9893	0.8164	0.2329
1	2014_11_11_FWW0070001	SVM	ALC	0.9798	0.5585	0.4415
1	2014_11_11_FWW0070001	DTSM	ALC	0.9738	0.4301	0.5727
1	2014_11_11_FWW0070001	Otsu	Otsu	0.9716	0.4026	0.6206
1	2014_11_11_FWW0070001	DTSM	HLC	0.9613	0.1538	0.8462
1	2014_11_11_FWW0070001	SVM	HLC	0.9543	0.0000	1.0000
1	2014_11_11_FWW0070001	Rowmeans	Rowmeans	0.2680	1.0000	16.0069
1	2014_11_18_FWW0070001	SVM	LLC	0.9837	0.9461	0.1022
1	2014_11_18_FWW0070001	DTSM	LLC	0.9815	0.9557	0.1159
1	2014_11_18_FWW0070001	SVM	ALC	0.9766	0.8654	0.1465
1	2014_11_18_FWW0070001	DTSM	ALC	0.9738	0.8709	0.1640
1	2014_11_18_FWW0070001	Otsu	Otsu	0.9693	0.9507	0.1925
1	2014_11_18_FWW0070001	DTSM	HLC	0.9621	0.8109	0.2376
1	2014_11_18_FWW0070001	K-means	K-means	0.9040	0.4027	0.6016
1	2014_11_18_FWW0070001	SVM	HLC	0.8784	0.2397	0.7617
1	2014_11_18_FWW0070001	Rowmeans	Rowmeans	0.2743	1.0000	4.5463
1	2014_11_25_FWW0070050	DTSM	LLC	0.9710	0.8391	0.1765
1	2014_11_25_FWW0070050	SVM	LLC	0.9697	0.8246	0.1842
1	2014_11_25_FWW0070050	Rowmeans	Rowmeans	0.9668	0.9343	0.2019
1	2014_11_25_FWW0070050	Otsu	Otsu	0.9419	0.9881	0.3532
1	2014_11_25_FWW0070050	SVM	ALC	0.9346	0.6063	0.3973
1	2014_11_25_FWW0070050	DTSM	ALC	0.9301	0.5814	0.4244
1	2014_11_25_FWW0070050	DTSM	HLC	0.9063	0.4434	0.5695
1	2014_11_25_FWW0070050	K-means	K-means	0.8907	0.3428	0.6638
1	2014_11_25_FWW0070050	SVM	HLC	0.8354	0.0000	1.0000
1	2014_11_28_FWW0070358	Otsu	Otsu	0.9866	0.9316	0.0974
1	2014_11_28_FWW0070358	Rowmeans	Rowmeans	0.9865	0.9305	0.0979

1	2014_11_28_FWW0070358	DTSM	LLC	0.9817	0.8732	0.1329
1	2014_11_28_FWW0070358	K-means	K-means	0.9805	0.8641	0.1410
1	2014_11_28_FWW0070358	SVM	LLC	0.9790	0.8502	0.1520
1	2014_11_28_FWW0070358	DTSM	HLC	0.9752	0.8232	0.1799
1	2014_11_28_FWW0070358	DTSM	ALC	0.9736	0.8100	0.1914
1	2014_11_28_FWW0070358	SVM	ALC	0.9675	0.7644	0.2357
1	2014_11_28_FWW0070358	SVM	HLC	0.9111	0.3557	0.6444
1	2014_12_02_FWW0070001	Rowmeans	Rowmeans	0.9785	0.9235	0.2123
1	2014_12_02_FWW0070001	K-means	K-means	0.9784	0.8319	0.2137
1	2014_12_02_FWW0070001	DTSM	LLC	0.9761	0.7808	0.2365
1	2014_12_02_FWW0070001	SVM	LLC	0.9753	0.7616	0.2440
1	2014_12_02_FWW0070001	Otsu	Otsu	0.9735	0.7845	0.2616
1	2014_12_02_FWW0070001	DTSM	ALC	0.9537	0.5537	0.4573
1	2014_12_02_FWW0070001	DTSM	HLC	0.9508	0.5289	0.4863
1	2014_12_02_FWW0070001	SVM	ALC	0.9497	0.5062	0.4966
1	2014_12_02_FWW0070001	SVM	HLC	0.9260	0.2747	0.7308
1	2014_12_05_FWW0070357	Rowmeans	Rowmeans	0.9833	0.9664	0.0999
1	2014_12_05_FWW0070357	Otsu	Otsu	0.9824	0.9351	0.1054
1	2014_12_05_FWW0070357	DTSM	LLC	0.9810	0.8919	0.1138
1	2014_12_05_FWW0070357	SVM	LLC	0.9784	0.8720	0.1296
1	2014_12_05_FWW0070357	DTSM	ALC	0.9662	0.7995	0.2027
1	2014_12_05_FWW0070357	DTSM	HLC	0.9639	0.7870	0.2162
1	2014_12_05_FWW0070357	SVM	ALC	0.9553	0.7324	0.2680
1	2014_12_05_FWW0070357	K-means	K-means	0.9113	0.4686	0.5318
1	2014_12_05_FWW0070357	SVM	HLC	0.8620	0.1729	0.8271
1	2014_12_09_FWW0070005	DTSM	LLC	0.9822	0.8844	0.1635
1	2014_12_09_FWW0070005	SVM	LLC	0.9811	0.8553	0.1729
1	2014_12_09_FWW0070005	SVM	ALC	0.9706	0.7401	0.2691
1	2014_12_09_FWW0070005	K-means	K-means	0.9680	0.8252	0.2934
1	2014_12_09_FWW0070005	DTSM	ALC	0.9635	0.6863	0.3345
1	2014_12_09_FWW0070005	DTSM	HLC	0.9485	0.5604	0.4720
1	2014_12_09_FWW0070005	SVM	HLC	0.8909	0.0000	1.0000
1	2014_12_09_FWW0070005	Otsu	Otsu	0.5273	0.9897	4.3316
1	2014_12_09_FWW0070005	Rowmeans	Rowmeans	0.3389	0.9948	6.0579
1	2014_12_09_FWW0070358	DTSM	LLC	0.9695	0.8195	0.1888
1	2014_12_09_FWW0070358	SVM	LLC	0.9660	0.7957	0.2104
1	2014_12_09_FWW0070358	Otsu	Otsu	0.9592	0.7761	0.2525
1	2014_12_09_FWW0070358	K-means	K-means	0.9563	0.7433	0.2709
1	2014_12_09_FWW0070358	Rowmeans	Rowmeans	0.9534	0.7322	0.2886
1	2014_12_09_FWW0070358	DTSM	ALC	0.9448	0.6610	0.3419
1	2014_12_09_FWW0070358	DTSM	HLC	0.9416	0.6458	0.3620
1	2014_12_09_FWW0070358	SVM	ALC	0.9411	0.6386	0.3647
1	2014_12_09_FWW0070358	SVM	HLC	0.8386	0.0000	1.0000
1	2014_12_16_FWW0070714	SVM	LLC	0.9762	0.9581	0.1138
1	2014_12_16_FWW0070714	DTSM	LLC	0.9748	0.9771	0.1206

1	2014_12_16_FWW0070714	DTSM	ALC	0.9715	0.9186	0.1364
1	2014_12_16_FWW0070714	SVM	ALC	0.9683	0.8785	0.1518
1	2014_12_16_FWW0070714	DTSM	HLC	0.9678	0.9276	0.1541
1	2014_12_16_FWW0070714	Rowmeans	Rowmeans	0.9515	0.8326	0.2322
1	2014_12_16_FWW0070714	Otsu	Otsu	0.9498	0.8233	0.2402
1	2014_12_16_FWW0070714	SVM	HLC	0.8852	0.4733	0.5495
1	2014_12_16_FWW0070714	K-means	K-means	0.8796	0.4256	0.5765
1	2015_01_09_FWW0070005	DTSM	LLC	0.9741	0.8024	0.2237
1	2015_01_09_FWW0070005	SVM	LLC	0.9724	0.7934	0.2383
1	2015_01_09_FWW0070005	K-means	K-means	0.9609	0.7576	0.3382
1	2015_01_09_FWW0070005	SVM	ALC	0.9563	0.6275	0.3780
1	2015_01_09_FWW0070005	DTSM	ALC	0.9348	0.4374	0.5635
1	2015_01_09_FWW0070005	DTSM	HLC	0.9290	0.4089	0.6138
1	2015_01_09_FWW0070005	Rowmeans	Rowmeans	0.9107	0.2352	0.7718
1	2015_01_09_FWW0070005	SVM	HLC	0.8844	0.0000	1.0000
1	2015_01_09_FWW0070005	Otsu	Otsu	0.8306	0.9581	1.4646
1	2015_01_15_FWW0070357	K-means	K-means	0.9769	0.8516	0.1790
1	2015_01_15_FWW0070357	DTSM	LLC	0.9738	0.8017	0.2034
1	2015_01_15_FWW0070357	SVM	LLC	0.9712	0.7814	0.2234
1	2015_01_15_FWW0070357	SVM	ALC	0.9551	0.6522	0.3483
1	2015_01_15_FWW0070357	DTSM	ALC	0.9520	0.6275	0.3728
1	2015_01_15_FWW0070357	DTSM	HLC	0.9429	0.5605	0.4432
1	2015_01_15_FWW0070357	Otsu	Otsu	0.9180	0.9980	0.6364
1	2015_01_15_FWW0070357	Rowmeans	Rowmeans	0.8711	0.0000	1.0000
1	2015_01_15_FWW0070357	SVM	HLC	0.8711	0.0000	1.0000
1	2015_03_05_FWW0070357	SVM	LLC	0.9714	0.7872	0.2520
1	2015_03_05_FWW0070357	DTSM	LLC	0.9685	0.7612	0.2776
1	2015_03_05_FWW0070357	SVM	ALC	0.9162	0.2625	0.7388
1	2015_03_05_FWW0070357	DTSM	HLC	0.9126	0.2409	0.7711
1	2015_03_05_FWW0070357	DTSM	ALC	0.9077	0.1865	0.8140
1	2015_03_05_FWW0070357	SVM	HLC	0.8866	0.0000	1.0000
1	2015_03_05_FWW0070357	K-means	K-means	0.8600	0.3630	1.2350
1	2015_03_05_FWW0070357	Rowmeans	Rowmeans	0.8495	0.2151	1.3273
1	2015_03_05_FWW0070357	Otsu	Otsu	0.1203	0.9048	7.7583
1	2015_03_23_FWW0070714	SVM	LLC	0.9591	0.7035	0.3093
1	2015_03_23_FWW0070714	DTSM	LLC	0.9560	0.6729	0.3329
1	2015_03_23_FWW0070714	Otsu	Otsu	0.9233	0.4630	0.5796
1	2015_03_23_FWW0070714	SVM	ALC	0.9182	0.3835	0.6186
1	2015_03_23_FWW0070714	K-means	K-means	0.9088	0.3143	0.6895
1	2015_03_23_FWW0070714	DTSM	HLC	0.9029	0.2665	0.7337
1	2015_03_23_FWW0070714	DTSM	ALC	0.9020	0.2597	0.7405
1	2015_03_23_FWW0070714	SVM	HLC	0.8773	0.0727	0.9273
1	2015_03_23_FWW0070714	Rowmeans	Rowmeans	0.8752	0.0564	0.9437
1	2015_03_26_FWW0070252	DTSM	LLC	0.9702	0.8102	0.1901
1	2015_03_26_FWW0070252	SVM	LLC	0.9693	0.8050	0.1963

1	2015_03_26_FWW0070252	K-means	K-means	0.9606	0.7615	0.2520
1	2015_03_26_FWW0070252	Otsu	Otsu	0.9592	0.7705	0.2604
1	2015_03_26_FWW0070252	SVM	ALC	0.9480	0.6676	0.3324
1	2015_03_26_FWW0070252	DTSM	ALC	0.9452	0.6502	0.3498
1	2015_03_26_FWW0070252	DTSM	HLC	0.9419	0.6287	0.3713
1	2015_03_26_FWW0070252	Rowmeans	Rowmeans	0.9408	0.6218	0.3782
1	2015_03_26_FWW0070252	SVM	HLC	0.8434	0.0000	1.0000
1	2015_03_30_FWW0070714	SVM	LLC	0.9742	0.8635	0.1397
1	2015_03_30_FWW0070714	DTSM	LLC	0.9737	0.8622	0.1426
1	2015_03_30_FWW0070714	Otsu	Otsu	0.9722	0.9735	0.1503
1	2015_03_30_FWW0070714	DTSM	ALC	0.9511	0.7372	0.2648
1	2015_03_30_FWW0070714	SVM	ALC	0.9504	0.7318	0.2684
1	2015_03_30_FWW0070714	DTSM	HLC	0.9485	0.7252	0.2785
1	2015_03_30_FWW0070714	K-means	K-means	0.9460	0.7118	0.2923
1	2015_03_30_FWW0070714	Rowmeans	Rowmeans	0.8445	0.1587	0.8415
1	2015_03_30_FWW0070714	SVM	HLC	0.8264	0.0602	0.9399
1	2015_04_16_FWW0070001	DTSM	LLC	0.9275	0.7963	0.2062
1	2015_04_16_FWW0070001	Otsu	Otsu	0.9263	0.8025	0.2098
1	2015_04_16_FWW0070001	SVM	LLC	0.9212	0.7775	0.2242
1	2015_04_16_FWW0070001	DTSM	HLC	0.9004	0.7201	0.2834
1	2015_04_16_FWW0070001	DTSM	ALC	0.8930	0.6962	0.3045
1	2015_04_16_FWW0070001	SVM	ALC	0.8853	0.6741	0.3264
1	2015_04_16_FWW0070001	K-means	K-means	0.8791	0.6567	0.3438
1	2015_04_16_FWW0070001	SVM	HLC	0.7247	0.2170	0.7831
1	2015_04_16_FWW0070001	Rowmeans	Rowmeans	0.7075	0.1679	0.8322
1	2015_04_27_FWW0070272	DTSM	LLC	0.9733	0.9402	0.0861
1	2015_04_27_FWW0070272	SVM	LLC	0.9716	0.9216	0.0915
1	2015_04_27_FWW0070272	DTSM	HLC	0.9705	0.9322	0.0951
1	2015_04_27_FWW0070272	DTSM	ALC	0.9702	0.9173	0.0960
1	2015_04_27_FWW0070272	Otsu	Otsu	0.9613	0.8865	0.1249
1	2015_04_27_FWW0070272	SVM	ALC	0.9601	0.8806	0.1287
1	2015_04_27_FWW0070272	SVM	HLC	0.8911	0.6515	0.3509
1	2015_04_27_FWW0070272	K-means	K-means	0.8851	0.6295	0.3705
1	2015_04_27_FWW0070272	Rowmeans	Rowmeans	0.7918	0.3287	0.6713
1	2015_04_30_FWW0070357	DTSM	LLC	0.9345	0.9003	0.1006
1	2015_04_30_FWW0070357	DTSM	HLC	0.9253	0.8881	0.1148
1	2015_04_30_FWW0070357	SVM	LLC	0.8996	0.8459	0.1544
1	2015_04_30_FWW0070357	DTSM	ALC	0.8892	0.8299	0.1704
1	2015_04_30_FWW0070357	Otsu	Otsu	0.8805	0.8221	0.1837
1	2015_04_30_FWW0070357	SVM	ALC	0.8716	0.8027	0.1973
1	2015_04_30_FWW0070357	K-means	K-means	0.7175	0.5657	0.4343
1	2015_04_30_FWW0070357	Rowmeans	Rowmeans	0.6933	0.5288	0.4714
1	2015_04_30_FWW0070357	SVM	HLC	0.6419	0.4496	0.5505
1	2015_05_04_FWW0070714	DTSM	LLC	0.9706	0.9429	0.0596
1	2015_05_04_FWW0070714	DTSM	HLC	0.9691	0.9493	0.0626

1	2015_05_04_FWW0070714	Otsu	Otsu	0.9665	0.9408	0.0681
1	2015_05_04_FWW0070714	DTSM	ALC	0.9495	0.8979	0.1026
1	2015_05_04_FWW0070714	SVM	LLC	0.9466	0.8930	0.1083
1	2015_05_04_FWW0070714	SVM	HLC	0.9194	0.8371	0.1636
1	2015_05_04_FWW0070714	SVM	ALC	0.9033	0.8037	0.1963
1	2015_05_04_FWW0070714	K-means	K-means	0.7970	0.5956	0.4121
1	2015_05_04_FWW0070714	Rowmeans	Rowmeans	0.5633	0.1136	0.8864
2	2014_11_07_FWW0070224	SVM	HLC	0.9812	0.5752	0.4927
2	2014_11_07_FWW0070224	K-means	K-means	0.9801	0.5056	0.5236
2	2014_11_07_FWW0070224	DTSM	ALC	0.9773	0.4173	0.5973
2	2014_11_07_FWW0070224	DTSM	HLC	0.9772	0.4202	0.5982
2	2014_11_07_FWW0070224	SVM	ALC	0.9017	0.7157	2.5827
2	2014_11_07_FWW0070224	SVM	LLC	0.7969	0.2276	5.3335
2	2014_11_07_FWW0070224	DTSM	LLC	0.7649	0.4431	6.1751
2	2014_11_07_FWW0070224	Otsu	Otsu	0.5795	0.9917	11.0429
2	2014_11_07_FWW0070224	Rowmeans	Rowmeans	0.5094	0.9971	12.8833
2	2014_11_07_FWW0070459	DTSM	ALC	0.9722	0.8015	0.3234
2	2014_11_07_FWW0070459	DTSM	HLC	0.9702	0.8212	0.3463
2	2014_11_07_FWW0070459	SVM	HLC	0.9694	0.8138	0.3552
2	2014_11_07_FWW0070459	SVM	ALC	0.9671	0.8648	0.3821
2	2014_11_07_FWW0070459	K-means	K-means	0.9577	0.6849	0.4921
2	2014_11_07_FWW0070459	SVM	LLC	0.9313	0.8338	0.7982
2	2014_11_07_FWW0070459	DTSM	LLC	0.9147	0.8600	0.9913
2	2014_11_07_FWW0070459	Otsu	Otsu	0.8034	0.9952	2.2856
2	2014_11_07_FWW0070459	Rowmeans	Rowmeans	0.6849	1.0000	3.6624
2	2014_12_12_FWW0070005	DTSM	ALC	0.9452	0.9564	0.2788
2	2014_12_12_FWW0070005	DTSM	HLC	0.9404	0.9638	0.3031
2	2014_12_12_FWW0070005	SVM	HLC	0.9050	0.9772	0.4829
2	2014_12_12_FWW0070005	Otsu	Otsu	0.8872	0.6344	0.5733
2	2014_12_12_FWW0070005	Rowmeans	Rowmeans	0.8851	0.5337	0.5841
2	2014_12_12_FWW0070005	K-means	K-means	0.8780	0.4774	0.6205
2	2014_12_12_FWW0070005	SVM	LLC	0.8369	0.9605	0.8295
2	2014_12_12_FWW0070005	DTSM	LLC	0.7938	0.9541	1.0482
2	2014_12_12_FWW0070005	SVM	ALC	0.7703	0.9166	1.1679
2	2014_12_12_FWW0070072	DTSM	ALC	0.9398	0.9427	0.2819
2	2014_12_12_FWW0070072	DTSM	HLC	0.9381	0.9552	0.2900
2	2014_12_12_FWW0070072	SVM	HLC	0.9148	0.9689	0.3989
2	2014_12_12_FWW0070072	Rowmeans	Rowmeans	0.8887	0.6505	0.5216
2	2014_12_12_FWW0070072	Otsu	Otsu	0.8871	0.6380	0.5287
2	2014_12_12_FWW0070072	K-means	K-means	0.8684	0.4779	0.6167
2	2014_12_12_FWW0070072	SVM	LLC	0.7898	0.9516	0.9848
2	2014_12_12_FWW0070072	DTSM	LLC	0.7561	0.9633	1.1425
2	2014_12_12_FWW0070072	SVM	ALC	0.7083	0.9332	1.3666
2	2014_12_19_FWW0070005	DTSM	ALC	0.9555	0.9194	0.1855
2	2014_12_19_FWW0070005	DTSM	HLC	0.9420	0.9354	0.2417

2	2014_12_19_FWW0070005	Otsu	Otsu	0.9303	0.8198	0.2903
2	2014_12_19_FWW0070005	K-means	K-means	0.9076	0.6529	0.3850
2	2014_12_19_FWW0070005	SVM	LLC	0.9048	0.8951	0.3966
2	2014_12_19_FWW0070005	Rowmeans	Rowmeans	0.8944	0.6009	0.4398
2	2014_12_19_FWW0070005	SVM	HLC	0.8833	0.9611	0.4862
2	2014_12_19_FWW0070005	DTSM	LLC	0.8805	0.9559	0.4978
2	2014_12_19_FWW0070005	SVM	ALC	0.6976	0.9798	1.2595
2	2014_12_19_FWW0070714	DTSM	ALC	0.9740	0.9035	0.2051
2	2014_12_19_FWW0070714	DTSM	HLC	0.9696	0.9110	0.2393
2	2014_12_19_FWW0070714	SVM	HLC	0.9651	0.9455	0.2754
2	2014_12_19_FWW0070714	Otsu	Otsu	0.9626	0.8121	0.2950
2	2014_12_19_FWW0070714	K-means	K-means	0.9520	0.6870	0.3780
2	2014_12_19_FWW0070714	Rowmeans	Rowmeans	0.9418	0.5870	0.4589
2	2014_12_19_FWW0070714	SVM	LLC	0.9365	0.9323	0.5004
2	2014_12_19_FWW0070714	SVM	ALC	0.9208	0.9683	0.6242
2	2014_12_19_FWW0070714	DTSM	LLC	0.9102	0.9394	0.7080
2	2014_12_23_FWW0070342	DTSM	ALC	0.9545	0.7638	0.2803
2	2014_12_23_FWW0070342	SVM	LLC	0.9543	0.8606	0.2819
2	2014_12_23_FWW0070342	DTSM	HLC	0.9520	0.7558	0.2956
2	2014_12_23_FWW0070342	K-means	K-means	0.9481	0.7370	0.3200
2	2014_12_23_FWW0070342	SVM	ALC	0.9467	0.8643	0.3284
2	2014_12_23_FWW0070342	DTSM	LLC	0.9424	0.8792	0.3552
2	2014_12_23_FWW0070342	SVM	HLC	0.9359	0.6636	0.3949
2	2014_12_23_FWW0070342	Rowmeans	Rowmeans	0.9189	0.5383	0.5001
2	2014_12_23_FWW0070342	Otsu	Otsu	0.8015	0.9733	1.2239
2	2014_12_23_FWW0070393	SVM	HLC	0.9601	0.8930	0.2031
2	2014_12_23_FWW0070393	DTSM	HLC	0.9573	0.8209	0.2170
2	2014_12_23_FWW0070393	Otsu	Otsu	0.9572	0.9182	0.2178
2	2014_12_23_FWW0070393	DTSM	ALC	0.9529	0.7905	0.2397
2	2014_12_23_FWW0070393	SVM	ALC	0.9527	0.9525	0.2407
2	2014_12_23_FWW0070393	DTSM	LLC	0.9371	0.8719	0.3200
2	2014_12_23_FWW0070393	Rowmeans	Rowmeans	0.9310	0.6773	0.3507
2	2014_12_23_FWW0070393	K-means	K-means	0.9282	0.6545	0.3651
2	2014_12_23_FWW0070393	SVM	LLC	0.9265	0.7662	0.3736
2	2015_01_13_FWW0070005	DTSM	HLC	0.9527	0.5843	0.4756
2	2015_01_13_FWW0070005	SVM	HLC	0.9484	0.6503	0.5198
2	2015_01_13_FWW0070005	DTSM	ALC	0.9466	0.5438	0.5377
2	2015_01_13_FWW0070005	K-means	K-means	0.9347	0.3505	0.6575
2	2015_01_13_FWW0070005	Rowmeans	Rowmeans	0.9337	0.5155	0.6671
2	2015_01_13_FWW0070005	SVM	LLC	0.9286	0.6431	0.7182
2	2015_01_13_FWW0070005	SVM	ALC	0.9250	0.7123	0.7551
2	2015_01_13_FWW0070005	DTSM	LLC	0.9235	0.6655	0.7701
2	2015_01_13_FWW0070005	Otsu	Otsu	0.9167	0.5743	0.8383
2	2015_01_13_FWW0070637	SVM	HLC	0.9728	0.8042	0.3182
2	2015_01_13_FWW0070637	DTSM	ALC	0.9697	0.7224	0.3540

2	2015_01_13_FWW0070637	Otsu	Otsu	0.9697	0.8236	0.3546
2	2015_01_13_FWW0070637	DTSM	HLC	0.9692	0.7342	0.3600
2	2015_01_13_FWW0070637	SVM	ALC	0.9683	0.8603	0.3702
2	2015_01_13_FWW0070637	K-means	K-means	0.9681	0.6966	0.3730
2	2015_01_13_FWW0070637	SVM	LLC	0.9608	0.8221	0.4577
2	2015_01_13_FWW0070637	DTSM	LLC	0.9276	0.8476	0.8459
2	2015_01_13_FWW0070637	Rowmeans	Rowmeans	0.6506	0.9642	4.0823
2	2015_03_09_FWW0070089	DTSM	HLC	0.8887	0.4851	0.5239
2	2015_03_09_FWW0070089	DTSM	LLC	0.8851	0.4917	0.5411
2	2015_03_09_FWW0070089	SVM	ALC	0.8824	0.4801	0.5538
2	2015_03_09_FWW0070089	SVM	LLC	0.8705	0.4004	0.6096
2	2015_03_09_FWW0070089	SVM	HLC	0.8691	0.4120	0.6162
2	2015_03_09_FWW0070089	DTSM	ALC	0.8666	0.3761	0.6279
2	2015_03_09_FWW0070089	K-means	K-means	0.8628	0.3619	0.6459
2	2015_03_09_FWW0070089	Otsu	Otsu	0.8615	0.6854	0.6519
2	2015_03_09_FWW0070089	Rowmeans	Rowmeans	0.8392	0.2511	0.7571
2	2015_03_09_FWW0070714	K-means	K-means	0.9597	0.7042	0.3244
2	2015_03_09_FWW0070714	DTSM	HLC	0.9577	0.6742	0.3409
2	2015_03_09_FWW0070714	SVM	ALC	0.9538	0.6645	0.3722
2	2015_03_09_FWW0070714	SVM	HLC	0.9471	0.6043	0.4260
2	2015_03_09_FWW0070714	Rowmeans	Rowmeans	0.9370	0.5111	0.5075
2	2015_03_09_FWW0070714	SVM	LLC	0.9338	0.4702	0.5328
2	2015_03_09_FWW0070714	DTSM	ALC	0.9308	0.4443	0.5571
2	2015_03_09_FWW0070714	DTSM	LLC	0.8812	0.6945	0.9568
2	2015_03_09_FWW0070714	Otsu	Otsu	0.1508	0.9999	6.8382
2	2015_03_12_FWW0070714	DTSM	HLC	0.9090	0.6552	0.3522
2	2015_03_12_FWW0070714	Otsu	Otsu	0.9050	0.6976	0.3679
2	2015_03_12_FWW0070714	K-means	K-means	0.9040	0.6471	0.3714
2	2015_03_12_FWW0070714	SVM	LLC	0.9032	0.6349	0.3747
2	2015_03_12_FWW0070714	DTSM	ALC	0.8822	0.5477	0.4560
2	2015_03_12_FWW0070714	SVM	ALC	0.8608	0.4722	0.5389
2	2015_03_12_FWW0070714	DTSM	LLC	0.8589	0.8243	0.5462
2	2015_03_12_FWW0070714	Rowmeans	Rowmeans	0.8358	0.3783	0.6356
2	2015_03_12_FWW0070714	SVM	HLC	0.8157	0.2925	0.7132
2	2015_03_16_FWW0070001	Otsu	Otsu	0.8627	0.3488	0.6982
2	2015_03_16_FWW0070001	K-means	K-means	0.8621	0.3305	0.7013
2	2015_03_16_FWW0070001	SVM	LLC	0.8599	0.3002	0.7125
2	2015_03_16_FWW0070001	Rowmeans	Rowmeans	0.8594	0.3089	0.7151
2	2015_03_16_FWW0070001	DTSM	HLC	0.8575	0.2853	0.7247
2	2015_03_16_FWW0070001	DTSM	ALC	0.8574	0.2837	0.7255
2	2015_03_16_FWW0070001	SVM	ALC	0.8528	0.2656	0.7489
2	2015_03_16_FWW0070001	SVM	HLC	0.8346	0.1667	0.8412
2	2015_03_16_FWW0070001	DTSM	LLC	0.8173	0.3539	0.9292
2	2015_03_19_FWW0070714	K-means	K-means	0.9622	0.7389	0.3083
2	2015_03_19_FWW0070714	Otsu	Otsu	0.9541	0.6818	0.3743

2	2015_03_19_FWW0070714	SVM	ALC	0.9143	0.3123	0.6981
2	2015_03_19_FWW0070714	Rowmeans	Rowmeans	0.9049	0.2296	0.7748
2	2015_03_19_FWW0070714	DTSM	HLC	0.9043	0.2230	0.7795
2	2015_03_19_FWW0070714	SVM	HLC	0.9021	0.2081	0.7976
2	2015_03_19_FWW0070714	DTSM	ALC	0.8979	0.1686	0.8317
2	2015_03_19_FWW0070714	SVM	LLC	0.8973	0.1637	0.8365
2	2015_03_19_FWW0070714	DTSM	LLC	0.8944	0.3086	0.8601
2	2015_04_07_FWW0070714	SVM	ALC	0.9471	0.8341	0.2341
2	2015_04_07_FWW0070714	DTSM	HLC	0.9414	0.7532	0.2593
2	2015_04_07_FWW0070714	Otsu	Otsu	0.9363	0.7761	0.2821
2	2015_04_07_FWW0070714	K-means	K-means	0.9261	0.6837	0.3272
2	2015_04_07_FWW0070714	SVM	HLC	0.9213	0.7459	0.3485
2	2015_04_07_FWW0070714	DTSM	ALC	0.9206	0.6529	0.3514
2	2015_04_07_FWW0070714	SVM	LLC	0.9019	0.5679	0.4344
2	2015_04_07_FWW0070714	DTSM	LLC	0.8615	0.7657	0.6134
2	2015_04_07_FWW0070714	Rowmeans	Rowmeans	0.8131	0.1744	0.8275
2	2015_04_09_FWW0070001	DTSM	HLC	0.9718	0.8398	0.1733
2	2015_04_09_FWW0070001	SVM	ALC	0.9709	0.8940	0.1791
2	2015_04_09_FWW0070001	SVM	HLC	0.9564	0.8182	0.2678
2	2015_04_09_FWW0070001	DTSM	ALC	0.9481	0.6863	0.3192
2	2015_04_09_FWW0070001	DTSM	LLC	0.9451	0.8226	0.3378
2	2015_04_09_FWW0070001	SVM	LLC	0.9308	0.5805	0.4254
2	2015_04_09_FWW0070001	Otsu	Otsu	0.9177	0.5125	0.5060
2	2015_04_09_FWW0070001	K-means	K-means	0.9052	0.4215	0.5827
2	2015_04_09_FWW0070001	Rowmeans	Rowmeans	0.9004	0.3969	0.6122
2	2015_04_13_FWW0070357	Otsu	Otsu	0.9341	0.8919	0.1657
2	2015_04_13_FWW0070357	SVM	ALC	0.8356	0.5962	0.4137
2	2015_04_13_FWW0070357	DTSM	HLC	0.7801	0.4476	0.5532
2	2015_04_13_FWW0070357	SVM	HLC	0.7731	0.4396	0.5709
2	2015_04_13_FWW0070357	DTSM	LLC	0.7678	0.4201	0.5843
2	2015_04_13_FWW0070357	K-means	K-means	0.7633	0.4567	0.5957
2	2015_04_13_FWW0070357	DTSM	ALC	0.7353	0.3342	0.6660
2	2015_04_13_FWW0070357	Rowmeans	Rowmeans	0.7321	0.3284	0.6740
2	2015_04_13_FWW0070357	SVM	LLC	0.7262	0.3113	0.6890
2	2015_04_20_FWW0070714	DTSM	LLC	0.9724	0.9315	0.1322
2	2015_04_20_FWW0070714	DTSM	HLC	0.9687	0.9100	0.1499
2	2015_04_20_FWW0070714	DTSM	ALC	0.9673	0.8615	0.1565
2	2015_04_20_FWW0070714	SVM	LLC	0.9495	0.7620	0.2419
2	2015_04_20_FWW0070714	Otsu	Otsu	0.9461	0.8115	0.2581
2	2015_04_20_FWW0070714	SVM	ALC	0.9315	0.9545	0.3280
2	2015_04_20_FWW0070714	K-means	K-means	0.9236	0.6394	0.3659
2	2015_04_20_FWW0070714	SVM	HLC	0.9186	0.9550	0.3900
2	2015_04_20_FWW0070714	Rowmeans	Rowmeans	0.7996	0.0402	0.9598
2	2015_04_23_FWW0070357	K-means	K-means	0.9645	0.9319	0.1249
2	2015_04_23_FWW0070357	Otsu	Otsu	0.9524	0.9699	0.1676

2	2015_04_23_FWW0070357	SVM	ALC	0.9307	0.7903	0.2438
2	2015_04_23_FWW0070357	DTSM	HLC	0.9270	0.7706	0.2570
2	2015_04_23_FWW0070357	DTSM	ALC	0.8940	0.6358	0.3729
2	2015_04_23_FWW0070357	SVM	HLC	0.8604	0.5303	0.4913
2	2015_04_23_FWW0070357	SVM	LLC	0.8347	0.4284	0.5818
2	2015_04_23_FWW0070357	Rowmeans	Rowmeans	0.7656	0.1768	0.8249
2	2015_04_23_FWW0070357	DTSM	LLC	0.7616	0.7956	0.8389
